# Do U.S. economic conditions at the state level predict the realized volatility of oil-price returns? A quantile machine-learning approach

**DOI:** 10.1186/s40854-022-00435-5

**Published:** 2023-01-12

**Authors:** Rangan Gupta, Christian Pierdzioch

**Affiliations:** 1 Department of Economics, University of Pretoria, Pretoria, 0002 South Africa; 2 Department of Economics, Helmut Schmidt University, Holstenhofweg 85, P.O.B. 700822, 22008 Hamburg, Germany

**Keywords:** Oil price, Realized volatility, Economic conditions indexes, Quantile Lasso, Prediction models, C22, C53, E32, E66, Q41

## Abstract

Because the U.S. is a major player in the international oil market, it is interesting to study whether aggregate and state-level economic conditions can predict the subsequent realized volatility of oil price returns. To address this research question, we frame our analysis in terms of variants of the popular heterogeneous autoregressive realized volatility (HAR-RV) model. To estimate the models, we use quantile-regression and quantile machine learning (Lasso) estimators. Our estimation results highlights the differential effects of economic conditions on the quantiles of the conditional distribution of realized volatility. Using weekly data for the period April 1987 to December 2021, we document evidence of predictability at a biweekly and monthly horizon.

## Introduction

In the wake of the severe global financial crisis (GFC) of 2007–2009 and a series of crises that followed, such as the European sovereign debt crisis, Brexit, and the ongoing COVID-19 pandemic, risks associated with portfolios comprising conventional financial assets have received considerable attention in recent empirical research (see, e.g., Balcilar et al. 2017a, 2020; Muteba Mwamba et al. 2017). However, because investors search for diversification opportunities, these crises have resulted in a noticeable trend towards alternative investment opportunities, including investments in commodities, in general, and oil, in particular (Bampinas and Panagiotidis 2015, 2017). This trend has led financial market participants to supplement their traditional portfolios with positions in commodities (Bahloul et al. 2018; Bonato 2019), and the resulting financilization of the commodity sector has been reflected in an increased participation of hedge funds, pension funds, and insurance companies in commodity markets. Crude oil is now considered a profitable alternative instrument in the portfolio decisions of financial institutions, implying that modeling and predicting the volatility of oil price movements has become a key issue in the financial industry and academic research (Degiannakis and Filis 2017). Considering this, the market size of crude-oil investments is $1.7 trillion per year at current spot prices, with 34 billion barrels produced each year and over 1.7 trillion barrels of crude oil in remaining reserves (U.S. Energy Information Administration (EIA); BP Statistical Review of World Energy), making it by far the most actively traded commodity.

The volatility of asset prices is an important input for investment decisions and portfolio choices; hence, accurate predictions of the volatility of oil price returns are of paramount importance to oil traders.^1^ Therefore, it is not surprising that a large and ever-burgeoning body of literature has considered the predictive value for the volatility of oil price returns of a large number of macroeconomic, financial, and behavioral variables, based on a wide spectrum of linear and non-linear models.^2^ Given this wide array of predictors, Guo et al. (2022) and Salisu et al. (2022) use the global economic conditions (GECON) index developed by Baumeister et al. (2020)^3^ to forecast the realized or conditional (generalized autoregressive conditional heteroskedasticity, i.e., GARCH) volatility of movements of the West Texas Intermediate (WTI) and Brent crude oil price, in addition to heating oil and natural gas, as well as exchange-traded funds (ETFs) of the global clean energy stock market (see also Wang et al. (2022) in this regard). These studies show that GECON, which is based on a set of 16 variables covering multiple dimensions of the global economy,^4^ outperforms the other popular predictors associated with global economic activity.^5^ Salisu et al. (2022) suggest that economic conditions are expected to affect oil price volatility based on the present-value model of asset prices (e.g., Shiller 1981a, b), given the financialization of commodity markets, whereby oil price return volatility depends on the volatility of cash flows and the discount factor (Conrad et al. 2014). In this regard, a worsening of global economic conditions (such as crisis periods) affects the volatility of variables that reflect future cash flows by generating economic uncertainty (Bernanke 1983) and the discount factor (Schwert 1989); hence, (a possibly negative) relationship between economic conditions and the volatility of oil price returns can be hypothesized.

Given the importance of global economic conditions in predicting the volatility of oil price returns, we extend this line of research by comparing the role of aggregate versus state-level metrics of economic conditions in the United States (U.S.) in predicting the subsequent realized volatility of WTI oil price returns over the weekly period from April 1987 to December 2021. In this regard, we rely on a novel dataset of weekly economic-condition indexes for the 50 U.S. states that cover multiple dimensions of the overall and state economies of the U.S.^6^ While the decision to analyze the predictive value of the aggregate U.S. economic conditions emanates from the works of Guo et al. (2022) and Salisu et al. (2022), the intuition to look at state-level economic conditions in predicting the realized volatility of oil price returns is straightforward, given the exceptional degree of heterogeneity at the state level in terms of oil dependency (calculated as oil consumed minus oil produced as a percentage of oil consumed). In the process, the strengths of their status as oil suppliers and demanders (De Michelis et al. 2020), as reflected by their underlying economic conditions. Understandably, if measures of state-level economic conditions produce better predictions relative to aggregate economic conditions, this finding is of considerable value to investors, as well as for academics, investigating the possibility of new factors that drive the volatility of oil price returns. Simultaneously, because the volatility of oil price returns has historically been shown to have predictive value for slowdowns in economic growth (van Eyden et al. 2019), policymakers can use relatively more precise estimates of future movements in the volatility of oil price returns to design macroeconomic policies ahead of time to prevent possible economic downturns. This could be achieved, for example, by feeding high-frequency predictions of the volatility of oil price returns into mixed data sampling (MIDAS) models associated with nowcasting of slow-moving, that is, low-frequency macroeconomic variables (Bańbura et al. 2011).

For our empirical research, from an econometric perspective, we use a machine-learning approach to analyze the predictive value of a large number of economic-conditions-based predictors associated with U.S. states. In particular, we rely on a quantiles-based version of the least absolute shrinkage and selection operator (Lasso) estimator (Tibshirani 1996). The idea underlying the Lasso estimator is to reduce the dimension of a predictive regression model in a data-driven manner to improve the interpretability of the model and the accuracy of predictions derived from the regularized model. However, rather than adhering to the standard linear Lasso estimator, we adopt a nonlinear setting and estimate the quantile-regression version of the Lasso estimator to study the predictive value of the economic conditions of the 50 states, in addition to a corresponding small-scale quantile-predictive regression model involving the overall U.S. economic conditions as a predictor. Pan et al. (2017) discuss the need to model nonlinearity in the relationship between the volatility of oil price returns and macroeconomic conditions . An advantage of our quantiles-based approach is that it enables us to develop a more complete characterization of the conditional distribution of the volatility of oil price returns through a set of conditional quantiles. A quantiles-based approach is more flexible than standard parametric approaches, such as linear regressions, Markov switching, and threshold regression models, and is robust to deviations from normality, including the presence of outliers (Gebka and Wohar 2019). Moreover, modeling only the conditional mean of the volatility of oil price returns through a linear or complex nonlinear regression model may hide interesting characteristics and lead us to conclude that predictors have poor predictive performance, while they are actually valuable for predicting certain quantiles of volatility (Gupta et al. 2017). In particular, our approach allows us to capture any potential asymmetric effect (nonlinear relationship) of economic conditions on the distribution of volatility, which renders it possible track different “types” of predictability.

At this stage, it is important to clarify two additional issues. First, we model the weekly realized volatility of returns of the WTI oil price, where we capture the realized volatility as the square root of the sum of daily squared returns over a week (following Andersen and Bollerslev 1998), which, in turn, yields an observable and unconditional measure of volatility, an otherwise latent process. Traditionally,^7^ researchers have studied the time-varying volatility of oil price returns using various models belonging to the GARCH family, under which conditional variance is a deterministic function of model parameters and past data. Alternatively, in recent studies, some researchers have considered stochastic volatility (SV) models, wherein volatility is depicted as a latent variable that follows a stochastic process. In this regard, whether a researcher uses GARCH or SV models, the resulting estimate of volatility is not unconditional (or model-free), as is the case with realized volatility. Second, while oil is a global commodity, because we focus on state-level economic conditions, we consider the WTI as our proxy for the world oil price. However, this should not be an issue, as the U.S. is a major player in both the demand and supply fronts of the oil market.

To the best of our knowledge, this is the first study to compare the role of aggregate and state-level measures of U.S. economic conditions to predict the realized volatility of oil price returns, using quantiles-based small-scale (involving only the national metric of economic conditions as a predictor) predictive regressions and a large-scale machine-learning quantile Lasso approach. By taking a regional versus aggregate perspective of economic conditions within the U.S., we build on the works of Guo et al. (2022) and Salisu et al. (2022), who focus on the role of global economic conditions in forecasting oil market volatility. The only other study that has analyzed the role of state-level variables in forecasting oil market volatility is that by Çepni et al. (2022), wherein the authors depict the importance of state-level uncertainty. Their study, however, is at a monthly frequency, unlike the weekly frequency in our case, which should be of more importance to investors and policymakers, in addition to dealing with a wide array of information capturing general economic conditions rather than just one aspect of regional economies, namely uncertainty. In other words, our study is more general than that of Çepni et al. (2022), especially when one realizes that the newspapers-based metrics of uncertainty employed by Çepni et al. (2022) may be endogenously driven by the economic conditions prevailing in the states (Mumtaz 2018; Mumtaz et al. 2018).

The remainder of our research is organized as follows. We describe our data in "Data" section, while we lay out our empirical methods in "Methods" section. We discuss our empirical results in "Empirical results" section, and conclude the paper in "Concluding remarks" section.

## Data

**Fig. 1:**
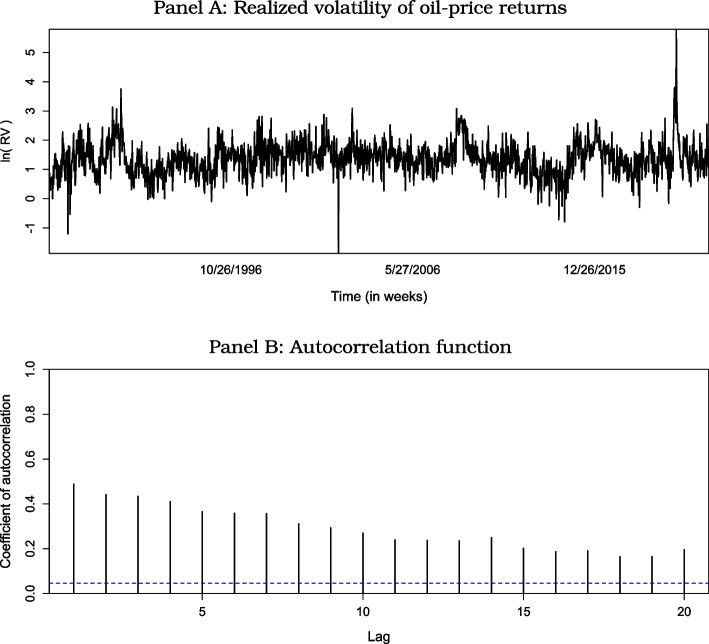
Properties of the Realized Volatility of Oil-Price Returns. Note: The dashed vertical line in Panel B denotes the 95% significance level

To construct our measure of the realized volatility (RV) of oil price returns, we first compute the daily log-returns (i.e., the first difference of the natural logarithm) of the West Texas Intermediate (WTI) oil price. In the second step, we compute the sum of the daily squared log returns over a specific week. In the third step, we obtain weekly realized volatility by taking the square root of this sum. The daily WTI crude oil nominal price data were derived from the Energy Information Administration (EIA) of the U.S.^8^ Because of the large peak in realized volatility at the end of the sample period, which is associated with the outbreak of the COVID-19 pandemic, we work with the (natural) logarithmic value of realized volatility. Working with log-realized volatility also avoids negativity issues and brings data closer to a normal distribution. Figure 1 plots the resulting time series of (log) realized volatility and its associated autocorrelation function. The slowly decaying pattern of the latter shows that the variants of the HAR-RV model that we lay out in detail in "Methods" section are natural candidates for studying the realized volatility of oil price returns.^9^

Regarding our main predictors, we analyze the role of the weekly economic-conditions indices (ECIs) of the overall U.S., as well as its 50 states. These indices are based on the work of Baumeister et al. (2022), who derive the indexes from mixed-frequency dynamic factor models with weekly, monthly, and quarterly variables that cover multiple dimensions of aggregate and state economies.^10^ Specifically, Baumeister et al. (2022) group variables into six broad categories: mobility measures, labor market indicators, real economic activity, expectations measures, financial indicators, and household indicators. Tables 8 and 9 at the end of the study (“Appendix”) provide details of the variables used in the construction of the weekly ECIs under each category at the state level and for the aggregate U.S., respectively. The indices are scaled to 4-quarter growth rates of U.S. real gross domestic product (GDP) and normalized such that a value of zero indicates national long-run growth.

Baumeister et al. (2022) find considerable cross-state heterogeneity in the length, depth, and timing of business cycles, which in turn provides a strong motivation to study the predictive value of not only aggregate but also state-level ECIs for the realized volatility of oil price returns. Based on data availability, our analysis covered the first week of April 1987 to the last week of December 2021.

## Methods

The heterogeneous autoregressive realized volatility (HAR-RV) model developed by Corsi (2009) is extensively used in earlier empirical research to study the realized volatility of oil price returns (see, for example, Degiannakis and Filis 2017; Gkillas et al. 2020a). Accordingly, we used the HAR-RV model as the nucleus in our predictive regression models. In the context of our empirical analysis, we formulate the HAR-RV model as follows:^11^

1 $$\begin{aligned} RV_{t+h} = \beta _0 + \beta _1 RV_t + \beta _2 RV_{bw,t} + \beta _3 RV_{m,t} + \epsilon _{t+h}, \end{aligned}$$

where $\epsilon _{t+h}$ denotes the disturbance term, *RV* denotes the realized weekly volatility of oil price returns, $RV_{bw,t}$ denotes the average biweekly *RV* from week $t - 2$ to week $t - 1$, and $RV_{m,t}$ denotes the average monthly *RV* from week $t - 4$ to week $t - 1$, with this structure motivated by the nature of the decay of the autocorrelation function of *RV* in Fig. 1. Parameter *h* denotes the horizon over which the subsequent realized volatility of oil price returns is studied. For $h > 1$, we compute $RV_{t+h}$ as the average realized volatility over the relevant horizon, where we study weekly ($h=1$), biweekly ($h= 2$), and monthly ($h = 4$) horizons. For example, in the case where $h= 2$, we have $RV_{t+h} = ( RV_{t+1} + RV_{t+2} ) / 2$. Equation (1) formalizes the basic idea behind the heterogeneous market hypothesis (Müller et al. 1997), according to which different groups of traders populate asset (commodity) markets, where traders belonging to the various groups differ with respect to their sensitivity to information flows at different time horizons.

As a first extension of the baseline model given in Eq. (1), we consider the possibility that aggregate economic conditions, *EC*,  in the U.S., a major player in the international oil market, may have predictive value for realized volatility. Therefore, we specify the HAR-RV-US model as follows:

2 $$\begin{aligned} RV_{t+h} = \beta _0 + \beta _1 RV_t + \beta _2 RV_{bw,t} + \beta _3 RV_{m,t} + \gamma ECI_{t, US} + \epsilon _{t+h}. \end{aligned}$$

As our second extension, we study a version of the baseline model that incorporates predictors, not the aggregate economic conditions in the U.S., but rather the economic conditions as measured at the level of individual states. This extension leads to the HAR-RV-states model:

3 $$\begin{aligned} RV_{t+h} = \beta _0 + \beta _1 RV_t + \beta _2 RV_{bw,t} + \beta _3 RV_{m,t} + \sum _{i=1}^{50} \gamma _i ECI_{t, i} + \epsilon _{t+h}, \end{aligned}$$

where index *i* denotes one of the 50 states. Given the large number of parameters of the HAR-RV-states model, it is preferable to estimate the predictive regression model given in Eq. (3) using parameter shrinkage and model regularization techniques.^12^ To this end, we used the least absolute shrinkage and selection operator (Lasso) proposed by Tibshirani (1996). The purpose of the Lasso estimator is to select a parsimonious version of the HAR-RV-states model by minimizing the following expression (see also the discussion in the textbook by Hastie et al. (2009)):

4 $$\begin{aligned} \sum _{t=1}^T \left( RV_{t+h} - \beta _0 - \beta _1 RV_t - \beta _2 RV_{bw,t} - \beta _3 RV_{m,t} - \sum _{i=1}^{50} \gamma _i ECI_{t, i} \right) ^2 + \lambda \left( \sum _{i=1}^{50} | \gamma _i | \right) \end{aligned}$$

where *T* denotes the number of observations and $\lambda$ denotes a shrinkage parameter. Equation (4) clarifies that the LASSO estimator adds to the standard quadratic loss function a penalty term that increases the absolute value of the coefficients to be estimated. Hence, the Lasso estimator implies that it is preferable to select coefficients that are small in absolute value or even zero, where the effect of model shrinkage must be balanced against its effect on the quadratic loss function. It should be noted that according to Eq. (4), we apply the Lasso model shrinkage only to shrink the coefficients of the states, not the intercept or coefficients of the classic HAR-RV model. The extent of shrinkage in the HAR-RV-states model depends on the magnitude of the shrinkage parameter. If the shrinkage parameter is sufficiently large, the Lasso estimator is set to zero for some or all coefficients. In our empirical research, we used tenfold cross-validation to optimize the value of the shrinkage parameter, where we used the check function to evaluate the cross-validated error.

A drawback of the predictive regression models given in Eqs. (1)–(4) is that they do not account for the possibility that the predictive value of economic activity for the subsequent realized volatility may depend on the quantile of the conditional distribution of the realized volatility oil price returns; that is, the predictive value of economic activity may depend on whether the oil market is in a state of low, intermediate, or high levels of volatility. To account for this possibility of nonlinearity, we study quantile-regression versions of the predictive regression models formalized in Eqs. (1)–(4) (see also Gkillas et al. (2020b), and Bonato et al. 2021, and for the seminal paper on quantile regressions, see Koenker and Bassett 1978). The quantile-regression versions of the HAR-RV model are given by

5 $$\begin{aligned} {\hat{\mathbf{b}}}_\alpha = \arg \min \sum _t^T \rho _\alpha \left( RV_{t+h} - \beta _{0,\alpha } - \beta _{1,\alpha } RV_t - \beta _{2,\alpha } RV_{bw,t} - \beta _{3,\alpha } RV_{m,t} \right) , \end{aligned}$$

where $\alpha$ denotes the quantile being studied, and ${\hat{\mathbf{b}}}_\alpha$ denotes the quantile-dependent vector of coefficients to be estimated (a hat denotes an estimated parameter). Function $\rho _\alpha$ is the check function, defined as $\rho _\alpha = \alpha \; \epsilon _{t+h}$ if $\epsilon _{t+h} > 0$ and $\rho _\alpha = ( \alpha - 1 ) \; \epsilon _{t+h}$ if $\epsilon _{t+h} < 0$. The quantile-regression version of the HAR-RV-US model can be derived by adding the aggregate U.S. economic activity to Eq. (2) as an additional predictor

The predictive regression model in Eq. (3) can be extended to a quantile-based predictive regression model in an analogous manner. However, given the large number of coefficients to be estimated, we do not estimate the quantile version of the HAR-RV-states model as a standard quantile-regression model, but rather as a penalized Lasso quantile-regression model (see Li and Zhu 2008; for a recent application of variants of the penalized quantile-regression techniques to a problem in energy economics, see Ren et al. 2022; for an analysis of the quantile Lasso approach in the context of a fixed-effects model (see also Koenker 2004). Accordingly, the quantile-regression version of the HAR-RV-states model is given by:

6 $$\begin{aligned} {\hat{\mathbf{b}}}_\alpha= & {} \arg \min \sum _t^T \rho _\alpha \left( RV_{t+h} - \beta _{0,\alpha } - \beta _{1,\alpha } RV_t - \beta _{2,\alpha } RV_{bw,t} - \beta _{3,\alpha } RV_{m,t} - \sum _{i=1}^{50} \gamma _{i, \alpha }ECI_{t, i} \right) \nonumber \\{} & {} + \lambda _\alpha \left( \sum _{i=1}^{50} | \gamma _{i, \alpha } | \right) \end{aligned}$$

where the shrinkage parameter was optimized given the quantile being analyzed.

To assess the fit of the various predictive regression models, we used a relative performance statistic (see also Koenker and Machado 1999; Pierdzioch et al. 2014, 2016). The relative performance *RP* is given by:

7 $$\begin{aligned} RP_{B, R | \alpha } = 1 - \frac{ \sum _{ t=1 }^T \rho _\alpha \left( e_{t, R} \right) }{ \sum _{ t=1 }^T \rho _\alpha \left( e_{t, B} \right) } \end{aligned}$$

where $e_{t,B}$ denotes the prediction error implied by the benchmark model and $e_{t,R}$ denotes the prediction error implied by the rival model. The summation in Eq. (7) runs over the entire sample when studying the full sample of the data. When we study the out-of-sample predictive values of the models, the summation runs over the relevant out-of-sample period.

It follows from the definition of the relative performance statistic given in Eq. (7) that given a quantile, the rival model performs better than the benchmark model when $RP > 0$. In turn, the benchmark model outperformed the rival model when $RP < 0$.^13^ It should be noted that, as is made explicit by Eq. (7), we evaluate the predictions under the same loss (check) function that we use to estimate the quantile (Lasso) regression models. Hence, as discussed by Koenker and Machado (1999), the relative performance statistic measures the relative predictive value of the benchmark and rival model at the quantile being studied in terms of a loss-function-weighted sum of absolute prediction errors. Therefore, the relative performance statistic is a quantile-specific local measure of relative predictive performance rather than a global measure evaluated over the entire conditional distribution of realized volatility. Such a local approach is a natural choice in the context of our empirical analysis because, as emphasized in "Introduction" section, we are interested in recovering the differential and potentially asymmetric effects of (state-level) economic conditions on different quantiles of the conditional distribution of realized volatility, rather than in inferring their global impact on predictive model performance over the entire conditional distribution.

We use the R language and environment for statistical computing (R Core Team 2021) to conduct our empirical research, where we use the R add-on package “rqPen” (Sherwood and Maidman 2020) to estimate the quantile (Lasso) regression models.

## Empirical results

Table 1 summarizes the baseline results. The table shows, for the three horizons being studied, the relative performance statistic, where we compare the classic HAR-RV model with the HAR-RV-US and HAR-RV-states models and the HAR-RV-US model with the HAR-RV-states model. Three main results were obtained: First, the relative performance statistics are close to zero for the weekly horizon, indicating that there are hardly any differences in the predictive values of the three models. This could be an indication that the information regarding the ECIs could not instantaneously impact demand and supply decisions in the oil market and took time to feed into oil price movements, as some production decisions were likely made ahead of time. Second, the relative performance statistic increases in the horizon when we compare the HAR-RV and HAR-RV-US models with the HAR-RV-states model. Hence, the incremental predictive value of state-level economic conditions strengthens in the biweekly and monthly horizons. This observation is in line with the one drawn above in terms of a time-lag, but it is also indicative of our initial motivation of investigating state-level ECIs, which allows us to better capture, relative to that of the overall economic conditions of the U.S., the demand- and supply-side dynamics of the oil market in line of the heterogeneity associated with oil dependence across the U.S. states. Third, accounting for state-level economic conditions at the biweekly and monthly horizons leverages relative performance, especially in the upper and lower quantiles of the conditional distribution of realized volatility. This effect was particularly pronounced in the monthly forecast horizon. Consequently, accounting for the impact of state-level economic conditions is especially useful for predicting the subsequent low and high realized volatility of oil price returns at the lower (5%) quantiles, especially at the upper (95%) quantiles.^14^ This result of detecting gains at extreme ends of oil market variability should not come as a surprise and can be explained following the works of Balcilar et al. (2017b) and Bonaccolto et al. (2018). In this regard, the median is indicative of normal levels of uncertainty prevailing in the oil market, and hence, does not require investors to utilize the information content of the ECIs for volatility. However, when the oil market is characterized by low or high degrees of volatility, it is understandable that oil market traders will want to use ECIs to predict where the future path of volatility is headed, that is, whether it is going to increase or decrease conditional on demand and supply conditions, so that they can make optimal portfolio decisions.

**Table 1:** Baseline results

Benchmark/rival model	h = 1	h = 2	h = 4
HAR-RV vs. HAR-RV-US/q = 0.95	0.0007	0.0015	0.0065
HAR-RV vs. HAR-RV-US/q = 0.75	0.0003	0.0000	0.0002
HAR-RV vs. HAR-RV-US/q = 0.5	0.0001	0.0001	0.0001
HAR-RV vs. HAR-RV-US/q = 0.25	0.0000	0.0002	0.0003
HAR-RV vs. HAR-RV-US/q = 0.05	0.0050	0.0015	0.0003
HAR-RV vs. HAR-RV-states/q = 0.95	0.0000	0.1113	0.1751
HAR-RV vs. HAR-RV-states/q = 0.75	0.0100	0.0719	0.1129
HAR-RV vs. HAR-RV-states/q = 0.5	0.0234	0.0370	0.0959
HAR-RV vs. HAR-RV-states/q = 0.25	0.0096	0.0578	0.0997
HAR-RV vs. HAR-RV-states/q = 0.05	0.0014	0.0504	0.1204
HAR-RV-US vs. HAR-RV-states/q = 0.95	− 0.0007	0.1100	0.1697
HAR-RV-US vs. HAR-RV-states/q = 0.75	0.0098	0.0719	0.1127
HAR-RV-US vs. HAR-RV-states/q = 0.5	0.0232	0.0369	0.0957
HAR-RV-US vs. HAR-RV-states/q = 0.25	0.0096	0.0576	0.0995
HAR-RV-US vs. HAR-RV-states/q = 0.05	− 0.0036	0.0489	0.1201

The relative-performance statistic, *RP*, statistic is computed as $RP = 1 - \sum _{t=1}^T \rho _\alpha \left( e_{t, R} \right) / \sum _{t=1}^T \rho _\alpha \left( e_{t, B} \right)$, where $e_t$ denotes the model prediction errors. The benchmark (B) model is the first model given in the first column of the table, and the rival (R) model is the second model given in that column. The HAR-RV-states model includes the state-level components in the vector of potential predictors. The benchmark model is estimated by the quantile-regression technique, while the HAR-RV-states model is estimated by the quantile Lasso technique. The intercept and the classic HAR-RV terms are not penalized. The penalty parameter is determined by tenfold cross-validation. A positive *RP* statistic shows that the rival model outperforms the benchmark model. The parameter *h* denotes the forecast horizon. The parameter *q* denotes the quantile being analyzed. The dependent variable is the natural log of the realized volatility of oil-price returns

Further results (not reported but available from the authors upon request) show that the average absolute size of the coefficients estimated for the various state-level economic conditions increases in the forecast horizon, especially at the monthly horizon. Moreover, at the monthly horizon, the average absolute size of the coefficients estimated for state-level economic conditions increases in the quantiles. Furthermore, the proportion of state-level economic conditions included in the penalized models increases as we move from the weekly to the monthly horizon. These three results should not come as a surprise, given the findings reported earlier, and indicate that economic conditions, especially for the states, gain importance over investment horizons, and are of more relevance to oil market players when uncertainty, that is, volatility in the oil market is already high, compared to situations where it is low or normal. In the weekly horizon, the proportion of state-level economic conditions included in the penalized models is relatively high (above 40%) at the median (which explains why the results of the permutation tests for the weekly horizon reported in Table 2 are significant at the median).^15^

The results of the permutation tests reported in Table 2 show that the increase in predictive performance resulting from extending the forecasting model to include state-level economic conditions to the vector of predictors is statistically significant, which is in line with our initial premise for the need for disaggregated information that can be derived from state-level ECIs. We implement the permutation tests as follows. We sample without replacement 500 times the state-level economic conditions. We then estimate the HAR-RV-states model using the quantile Lasso estimator on the simulated data and store the model prediction errors. Next, we compute the relative performance statistics for every simulated dataset, where the prediction errors of the benchmark HAR-RV-US model are based on the estimates reported in Table 1. Finally, we compute the *p* value of the permutation test as the proportion of the relative performance statistics computed for the simulated data, which exceeds the relative performance statistics reported in Table 1. If the state-level economic conditions contribute to the predictive performance of the model, the simulated relative performance statistics should fall short of the relative performance statistics documented in Table 1 most of the time.

**Table 2:** Results of permutation tests

Benchmark/rival model	h = 1	h = 2	h = 4
HAR-RV-US vs. HAR-RV-states/q = 0.95	0.7680	0.0000	0.0000
HAR-RV-US vs. HAR-RV-states/q = 0.75	0.0980	0.0000	0.0000
HAR-RV-US vs. HAR-RV-states/q = 0.5	0.0000	0.0000	0.0000
HAR-RV-US vs. HAR-RV-states/q = 0.25	0.1220	0.0000	0.0000
HAR-RV-US vs. HAR-RV-states/q = 0.05	0.5540	0.0000	0.0000

The *p* values reported in this table are based on 500 simulation runs. In every simulation run, the data on the state-level economic conditions are sampled without replacement. The dependent variable and the predictors of the classic HAR-RV model are not resampled. Using the simulated data, the HAR-RV-states model is then estimated by means of the quantile Lasso estimator (the intercept and the classic HAR-RV terms are not penalized) and the model prediction errors are stored. The penalty parameter is determined by tenfold cross-validation. The relative-performance statistic, *RP*, statistic is computed as $RP = 1 - \sum _{t=1}^T \rho _\alpha \left( e_{t, R} \right) / \sum _{t=1}^T \rho _\alpha \left( e_{t, B} \right)$, where *i* denotes the simulation index, $e_t$ denotes the model prediction errors, B denotes the benchmark model, and R denotes the rival model. The benchmark (rival) model is the first (second) model given in the first column of the table. The benchmark model is estimated by the quantile-regression technique. The prediction errors of the benchmark model are based on the estimates reported in Table 1. The *p* values are then computed as $( \# RP_i \ge RP_{ref} ) / 500$, where the reference values, $RP_{ref}$, of the relative performance statistic are the values reported in Table 1. The parameter *h* denotes the forecast horizon. The parameter *q* denotes the quantile being analyzed. The dependent variable is the natural log of the realized volatility of oil-price returns

The results of the permutation tests show that at the weekly horizon, predictive performance due to state-level economic conditions increases in a statistically significant way, mainly at the median. At the biweekly and monthly forecast horizons, all the permutation tests yielded significant results. In other words, we find strong evidence that state-level economic conditions help to improve in a statistically significant way predictions of the subsequent realized volatility of oil-price returns at the biweekly and monthly horizons. This finding supports the basic motivation of looking at state-level economic conditions in addition to the overall condition, as we expect the former to better capture the demand and supply of oil, particularly as the forecast horizon increases, by accounting for heterogenous oil dependency across the states.

**Table 3:** Results for RV

Benchmark/rival model	h = 1	h = 2	h = 4
HAR-RV vs. HAR-RV-US/q = 0.95	0.0000	0.0000	0.0003
HAR-RV vs. HAR-RV-US/q = 0.75	0.0002	0.0002	0.0002
HAR-RV vs. HAR-RV-US/q = 0.5	0.0000	0.0003	0.0002
HAR-RV vs. HAR-RV-US/q = 0.25	0.0016	0.0009	0.0000
HAR-RV vs. HAR-RV-US/q = 0.05	0.0031	0.0000	0.0002
HAR-RV vs. HAR-RV-states/q = 0.95	0.0002	0.0000	0.2340
HAR-RV vs. HAR-RV-states/q = 0.75	0.0317	0.0521	0.0809
HAR-RV vs. HAR-RV-states/q = 0.5	0.0426	0.0688	0.0913
HAR-RV vs. HAR-RV-states/q = 0.25	0.0598	0.1023	0.1425
HAR-RV vs. HAR-RV-states/q = 0.05	0.0414	0.1334	0.2129
HAR-RV-US vs. HAR-RV-states/q = 0.95	0.0002	0.0000	0.2337
HAR-RV-US vs. HAR-RV-states/q = 0.75	0.0315	0.0518	0.0807
HAR-RV-US vs. HAR-RV-states/q = 0.5	0.0426	0.0686	0.0911
HAR-RV-US vs. HAR-RV-states/q = 0.25	0.0583	0.1014	0.1425
HAR-RV-US vs. HAR-RV-states/q = 0.05	0.0384	0.1334	0.2127

The relative-performance statistic, *RP*, statistic is computed as $RP = 1 - \sum _{t=1}^T \rho _\alpha \left( e_{t, R} \right) / \sum _{t=1}^T \rho _\alpha \left( e_{t, B} \right)$, where $e_t$ denotes the model prediction errors. The benchmark (B) model is the first model given in the first column of the table, and the rival (R) model is the second model given in that column. The HAR-RV-states model includes the state-level components in the vector of potential predictors. The benchmark model is estimated by the quantile-regression technique, while the HAR-RV-states model is estimated by the quantile Lasso technique. The intercept and the classic HAR-RV terms are not penalized. The penalty parameter is determined by tenfold cross-validation. A positive *RP* statistic shows that the rival model outperforms the benchmark model. The parameter *h* denotes the forecast horizon. The parameter *q* denotes the quantile being analyzed. The dependent variable is the realized volatility of oil-price returns

Next, we report the robustness check results for realized volatility (rather than its logarithm) in Table 3. There were no changes in the general picture. The HAR-RV-US model does not add predictive value over and above the predictive value of the classic HAR-RV model, accounting for state-level economic conditions, boosts relative predictive performance, especially at the biweekly and monthly horizons. Moreover, the impact of state-level economic conditions on relative performance in the monthly horizon is again strongest in the lower and upper quantiles. These findings are in line with the underlying intuition presented above in terms of time lags, heterogeneity of oil dependency, market states affecting investment decisions, and the fact that it remains consistent irrespective of the scaling of the process of volatility, confirming the robustness of our understanding of how oil market volatility is affected by economic conditions, even though we are using an atheoretical approach here to forecast oil realized volatility.

**Table 4:** Results of an out-of-sample analysis

Benchmark/rival model	Mean RP	*p* value	Mean RP	*p* value	Mean RP	*p* value
Horizon	h = 1	h = 1	h = 2	h = 2	h = 4	h = 4
HAR-RV vs. HAR-RV-US/q = 0.95	− 0.0019	0.5920	− 0.0030	0.5160	0.0040	0.3000
HAR-RV vs. HAR-RV-US/q = 0.75	− 0.0005	0.5040	− 0.0011	0.7760	− 0.0009	0.6060
HAR-RV vs. HAR-RV-US/q = 0.5	− 0.0007	0.6720	− 0.0005	0.6860	− 0.0011	0.7300
HAR-RV vs. HAR-RV-US/q = 0.25	− 0.0010	0.7480	− 0.0008	0.5980	− 0.0009	0.5700
HAR-RV vs. HAR-RV-US/q = 0.05	0.0024	0.3300	− 0.0020	0.4980	− 0.0026	0.6920
HAR-RV vs. HAR-RV-states/q = 0.95	− 0.0093	0.7380	− 0.0143	0.6560	0.0646	0.0780
HAR-RV vs. HAR-RV-states/q = 0.75	− 0.0033	0.5840	0.0252	0.0600	0.0466	0.0200
HAR-RV vs. HAR-RV-states/q = 0.5	0.0020	0.2940	0.0074	0.2220	0.0507	0.0020
HAR-RV vs. HAR-RV-states/q = 0.25	− 0.0043	0.6660	0.0132	0.1540	0.0465	0.0060
HAR-RV vs. HAR-RV-states/q = 0.05	− 0.0047	0.6520	0.0049	0.3280	0.0443	0.0660
HAR-RV-US vs. HAR-RV-states/q = 0.95	− 0.0073	0.5500	− 0.0113	0.5780	0.0608	0.0840
HAR-RV-US vs. HAR-RV-states/q = 0.75	− 0.0028	0.5120	0.0263	0.0560	0.0474	0.0160
HAR-RV-US vs. HAR-RV-states/q = 0.5	0.0027	0.2600	0.0080	0.2060	0.0517	0.0020
HAR-RV-US vs. HAR-RV-states/q = 0.25	− 0.0032	0.5100	0.0140	0.1340	0.0474	0.0060
HAR-RV-US vs. HAR-RV-states/q = 0.05	− 0.0071	0.7280	0.0068	0.3040	0.0467	0.0620

The models are estimated 500 times on bootstrapped data sampled without replacement. For every estimation, the relative-performance statistic, *RP*, statistic is computed as $RP = 1 - \sum _{t=1}^T \rho _\alpha \left( e_{t, R} \right) / \sum _{t=1}^T \rho _\alpha \left( e_{t, B} \right)$, where $e_t$ denotes the out-of-sample prediction errors and the summation, $t,,\ldots , T$, runs over the out-of-sample data. The out-of-sample data are those data not included in the estimation. The fraction of out-of-sample data for every bootstrap sample is 30%. The benchmark (B) model is the first model given in the first column of the table, and the rival (R) model is the second model given in that column. The HAR-RV-states model includes the state-level components in the vector of potential predictors. The benchmark model is estimated by the quantile-regression technique, while the HAR-RV-states model is estimated by the quantile Lasso technique. The intercept and the classic HAR-RV terms are not penalized. The penalty parameter is determined by tenfold cross-validation and is re-optimized at the beginning of a month. A positive mean of the out-of-sample *RP* statistic shows that the rival model outperforms on average the benchmark model. The parameter *h* denotes the forecast horizon. The parameter *q* denotes the quantile being analyzed. The dependent variable is the natural log of the realized volatility of oil-price returns

It is also interesting to analyze predictive performance in a quasi-out-of-sample context. To this end, we bootstrap the data 500 times without replacement, fixing the fraction of out-of-sample data for every bootstrap sample at 30%. We then estimate all three models on the bootstrapped data and make forecasts of the “out-of-sample data” (also known as the out-of-bag data in the machine-learning literature; it should be noted that sampling without replacement implies that the out-of-bag data are not included in the sample of data on which we train the model). For every bootstrap sample, we compute the relevant relative performance statistics. Finally, we compute the mean of the resulting sampling distributions of the relative performance statistics and study the proportion of negative relative performance statistics (which indicates that the benchmark model is superior to the rival model). We document the results in Table 4. As expected, the performance statistics were smaller than those summarized in Table 1. At the weekly horizon, the relative performance statistics are negative or close to zero, on average, for all three model combinations. Not surprisingly, the *p* values demonstrate that neither the HAR-RV-US nor the HAR-RV-states model exceeds the HAR-RV model in terms of predictive value. At the biweekly horizon, while the relative performance statistics for the HAR-RV-US remain negative on average, the mean values of the relative performance statistics for the HAR-RV-states model mostly take a positive but small value. There is some evidence that accounting for state-level economic conditions helps significantly increase predictive performance for the 75% quantile. Finally, for the monthly horizon, the *p* values for the HAR-RV-US model remain well above conventional significance levels, but the *p* values for the HAR-RV-states model show that state-level economic conditions significantly boost the predictive performance for all five quantiles being studied. Hence, we find evidence of the ability of state-level economic conditions, as with the in-sample tests, to predict gains for oil market volatility, particularly in the medium (bi) to the long run. While these findings can benefit oil market investors in their portfolio decisions, they tend to corroborate our underlying explanation of the in-sample results discussed above, especially with time lags and oil dependency across states.

**Table 5:** Results for components of state-level economic conditions

Benchmark/rival model	h = 1	h = 2	h = 4
	*Expectations*		
HAR-RV vs. HAR-RV-states/q = 0.95	0.0218	0.1292	0.1412
HAR-RV vs. HAR-RV-states/q = 0.75	0.0205	0.0654	0.0833
HAR-RV vs. HAR-RV-states/q = 0.5	0.0154	0.0390	0.0733
HAR-RV vs. HAR-RV-states/q = 0.25	0.0030	0.0186	0.0732
HAR-RV vs. HAR-RV-states/q = 0.05	0.0052	0.0341	0.0749
HAR-RV-US vs. HAR-RV-states/q = 0.95	0.0211	0.1279	0.1356
HAR-RV-US vs. HAR-RV-states/q = 0.75	0.0202	0.0654	0.0832
HAR-RV-US vs. HAR-RV-states/q = 0.5	0.0153	0.0389	0.0732
HAR-RV-US vs. HAR-RV-states/q = 0.25	0.0030	0.0184	0.0729
HAR-RV-US vs. HAR-RV-states/q = 0.05	0.0002	0.0326	0.0746
	*Financials*		
HAR-RV vs. HAR-RV-states/q = 0.95	0.0416	0.1628	0.2245
HAR-RV vs. HAR-RV-states/q = 0.75	0.0490	0.0833	0.1458
HAR-RV vs. HAR-RV-states/q = 0.5	0.0271	0.0580	0.1083
HAR-RV vs. HAR-RV-states/q = 0.25	0.0017	0.0577	0.1209
HAR-RV vs. HAR-RV-states/q = 0.05	0.0267	0.1026	0.1075
HAR-RV-US vs. HAR-RV-states/q = 0.95	0.0410	0.1616	0.2195
HAR-RV-US vs. HAR-RV-states/q = 0.75	0.0487	0.0833	0.1456
HAR-RV-US vs. HAR-RV-states/q = 0.5	0.0270	0.0579	0.1081
HAR-RV-US vs. HAR-RV-states/q = 0.25	0.0017	0.0575	0.1206
HAR-RV-US vs. HAR-RV-states/q = 0.05	0.0218	0.1012	0.1072
	*Households*		
HAR-RV vs. HAR-RV-states/q = 0.95	0.0000	0.1586	0.1788
HAR-RV vs. HAR-RV-states/q = 0.75	0.0128	0.0844	0.1204
HAR-RV vs. HAR-RV-states/q = 0.5	0.0208	0.0434	0.1010
HAR-RV vs. HAR-RV-states/q = 0.25	0.0386	0.0457	0.0947
HAR-RV vs. HAR-RV-states/q = 0.05	0.0349	0.0556	0.1232
HAR-RV-US vs. HAR-RV-states/q = 0.95	− 0.0007	0.1574	0.1734
HAR-RV-US vs. HAR-RV-states/q = 0.75	0.0126	0.0844	0.1203
HAR-RV-US vs. HAR-RV-states/q = 0.5	0.0206	0.0433	0.1009
HAR-RV-US vs. HAR-RV-states/q = 0.25	0.0386	0.0455	0.0944
HAR-RV-US vs. HAR-RV-states/q = 0.05	0.0300	0.0541	0.1229
	*Labour market*		
HAR-RV vs. HAR-RV-states/q = 0.95	0.0000	0.1580	0.2217
HAR-RV vs. HAR-RV-states/q = 0.75	0.0000	0.0801	0.1318
HAR-RV vs. HAR-RV-states/q = 0.5	0.0279	0.0254	0.1002
HAR-RV vs. HAR-RV-states/q = 0.25	0.0364	0.0277	0.1013
HAR-RV vs. HAR-RV-states/q = 0.05	0.0307	0.0457	0.1295
HAR-RV-US vs. HAR-RV-states/q = 0.95	− 0.0007	0.1568	0.2166
HAR-RV-US vs. HAR-RV-states/q = 0.75	− 0.0003	0.0801	0.1317
HAR-RV-US vs. HAR-RV-states/q = 0.5	0.0278	0.0252	0.1001
HAR-RV-US vs. HAR-RV-states/q = 0.25	0.0364	0.0275	0.1010
HAR-RV-US vs. HAR-RV-states/q = 0.05	0.0258	0.0442	0.1292
	*Mobility*		
HAR-RV vs. HAR-RV-states/q = 0.95	0.0060	0.1409	0.2098
HAR-RV vs. HAR-RV-states/q = 0.75	0.0253	0.0660	0.1128
HAR-RV vs. HAR-RV-states/q = 0.5	0.0295	0.0249	0.0784
HAR-RV vs. HAR-RV-states/q = 0.25	0.0012	0.0427	0.0671
HAR-RV vs. HAR-RV-states/q = 0.05	0.0001	0.0947	0.1317
HAR-RV-US vs. HAR-RV-states/q = 0.95	0.0053	0.1396	0.2047
HAR-RV-US vs. HAR-RV-states/q = 0.75	0.0251	0.0659	0.1126
HAR-RV-US vs. HAR-RV-states/q = 0.5	0.0294	0.0247	0.0783
HAR-RV-US vs. HAR-RV-states/q = 0.25	0.0012	0.0425	0.0668
HAR-RV-US vs. HAR-RV-states/q = 0.05	− 0.0049	0.0933	0.1314
	*Real activity*		
HAR-RV vs. HAR-RV-states/q = 0.95	0.0280	0.1327	0.2037
HAR-RV vs. HAR-RV-states/q = 0.75	0.0219	0.0684	0.1026
HAR-RV vs. HAR-RV-states/q = 0.5	0.0193	0.0353	0.0892
HAR-RV vs. HAR-RV-states/q = 0.25	0.0000	0.0436	0.0829
HAR-RV vs. HAR-RV-states/q = 0.05	0.0000	0.0601	0.1284
HAR-RV-US vs. HAR-RV-states/q = 0.95	0.0273	0.1314	0.1985
HAR-RV-US vs. HAR-RV-states/q = 0.75	0.0216	0.0684	0.1025
HAR-RV-US vs. HAR-RV-states/q = 0.5	0.0192	0.0352	0.0891
HAR-RV-US vs. HAR-RV-states/q = 0.25	0.0000	0.0434	0.0826
HAR-RV-US vs. HAR-RV-states/q = 0.05	− 0.0050	0.0587	0.1281

The relative-performance statistic, *RP*, statistic is computed as $RP = 1 - \sum _{t=1}^T \rho _\alpha \left( e_{t, R} \right) / \sum _{t=1}^T \rho _\alpha \left( e_{t, B} \right)$, where $e_t$ denotes the model prediction errors. The benchmark (B) model is the first model given in the first column of the table, and the rival (R) model is the second model given in that column. The HAR-RV-states model includes the state-level components in the vector of potential predictors. The benchmark model is estimated by the quantile-regression technique, while the HAR-RV-states model is estimated by the quantile Lasso technique. The intercept and the classic HAR-RV terms are not penalized. The penalty parameter is determined by tenfold cross-validation. A positive *RP* statistic shows that the rival model outperforms the benchmark model. The parameter *h* denotes the forecast horizon. The parameter *q* denotes the quantile being analyzed. The dependent variable is the natural log of the realized volatility of oil-price returns

In Table 5, we document that the results that we find for state-level economic conditions also hold when we study the components of state-level economic condition indexes (expectations, financials, households, labor market, mobility, and real activity).^16^ The components of state-level economic conditions contribute to predictive performance (relative to the HAR-RV and HAR-RV-US models) mainly at the biweekly and monthly horizons and at the lower and, especially, at the upper (95%) quantiles, demonstrating the robustness of our results. In other words, the use of the overall ECIs of the states can convey the same information that can be obtained from its disaggregated component. This implies that the usage of all underlying information that goes in the construction of state-level ECIs is important, whether in an aggregate manner or with the separate components considered simultaneously, indicating the importance of the various economic categories of variables considered in appropriately capturing the price dynamics of the oil market.

**Table 6:** Results for the weakness index

Benchmark/rival model	h = 1	h = 2	h = 4
HAR-RV vs. HAR-RV-weak/q = 0.95	0.0012	0.0020	0.0027
HAR-RV vs. HAR-RV-weak/q = 0.75	0.0002	0.0000	0.0000
HAR-RV vs. HAR-RV-weak/q = 0.5	0.0001	0.0001	0.0000
HAR-RV vs. HAR-RV-weak/q = 0.25	0.0000	0.0005	0.0010
HAR-RV vs. HAR-RV-weak/q = 0.05	0.0054	0.0017	0.0020
HAR-RV vs. HAR-RV-states/q = 0.95	0.0052	0.1146	0.1841
HAR-RV vs. HAR-RV-states/q = 0.75	0.0093	0.0706	0.1131
HAR-RV vs. HAR-RV-states/q = 0.5	0.0234	0.0554	0.0956
HAR-RV vs. HAR-RV-states/q = 0.25	0.0105	0.0527	0.0954
HAR-RV vs. HAR-RV-states/q = 0.05	0.0311	0.0463	0.1014
HAR-RV-weak vs. HAR-RV-states/q = 0.95	0.0040	0.1129	0.1819
HAR-RV-weak vs. HAR-RV-states/q = 0.75	0.0091	0.0706	0.1131
HAR-RV-weak vs. HAR-RV-states/q = 0.5	0.0233	0.0553	0.0955
HAR-RV-weak vs. HAR-RV-states/q = 0.25	0.0105	0.0523	0.0945
HAR-RV-weak vs. HAR-RV-states/q = 0.05	0.0259	0.0447	0.0996

The relative-performance statistic, *RP*, statistic is computed as $RP = 1 - \sum _{t=1}^T \rho _\alpha \left( e_{t, R} \right) / \sum _{t=1}^T \rho _\alpha \left( e_{t, B} \right)$, where $e_t$ denotes the model prediction errors. The benchmark (B) model is the first model given in the first column of the table, and the rival (R) model is the second model given in that column The HAR-RV-states model includes the state-level components in the vector of potential predictors. The benchmark model is estimated by the quantile-regression technique, while the HAR-RV-states model is estimated by the quantile Lasso technique. The intercept and the classic HAR-RV terms are not penalized. The penalty parameter is determined by tenfold cross-validation. A positive *RP* statistic shows that the rival model outperforms the benchmark model. The parameter *h* denotes the forecast horizon. The parameter *q* denotes the quantile being analyzed. The dependent variable is the natural log of the realized volatility of oil-price returns

As a further illustration of the robustness of our results, we report in Table 6 the results that we obtained when we replaced the data on the economic conditions index of the overall U.S. with the economic weakness index (EWI).^17^ The EWI is a summary measure of national business cycle dynamics and is constructed using state-level recession probabilities extracted from a Markov-switching model that allows for heterogeneous recessions and expansions (see Baumeister et al. 2022, for further details). The general pattern of our results remained unchanged. State-level economic activity again contributes to the predictive performance at the biweekly and monthly horizons, where this contribution is particularly strong at the upper quantile of the conditional distribution of the realized volatility of oil price returns. Hence, we can safely say that our economic explanation for the obtained econometric results is not sensitive to the choice of the metric of economic conditions involving the entire U.S., which again highlights the importance of the economic conditions at the state level in better capturing the underlying heterogenous nature of demand and supply of oil.

**Table 7:** Additional results based on intraday data calculations

Benchmark/rival model	h = 1	h = 2	h = 4
	*Crude oil*		
HAR-RV vs. HAR-RV-US/q = 0.95	0.0000	0.0000	0.0003
HAR-RV vs. HAR-RV-US/q = 0.75	0.0001	0.0001	0.0003
HAR-RV vs. HAR-RV-US/q = 0.5	0.0003	0.0002	0.0000
HAR-RV vs. HAR-RV-US/q = 0.25	0.0000	0.0000	0.0001
HAR-RV vs. HAR-RV-US/q = 0.05	0.0007	0.0019	0.0005
HAR-RV vs. HAR-RV-states/q = 0.95	0.0068	0.1771	0.1184
HAR-RV vs. HAR-RV-states/q = 0.75	0.0042	0.0729	0.1275
HAR-RV vs. HAR-RV-states/q = 0.5	0.0101	0.0528	0.1050
HAR-RV vs. HAR-RV-states/q = 0.25	0.0347	0.0561	0.0938
HAR-RV vs. HAR-RV-states/q = 0.05	0.1175	0.1902	0.2544
HAR-RV-US vs. HAR-RV-states/q = 0.95	0.0068	0.1771	0.1182
HAR-RV-US vs. HAR-RV-states/q = 0.75	0.0041	0.0729	0.1272
HAR-RV-US vs. HAR-RV-states/q = 0.5	0.0098	0.0527	0.1050
HAR-RV-US vs. HAR-RV-states/q = 0.25	0.0346	0.0561	0.0938
HAR-RV-US vs. HAR-RV-states/q = 0.05	0.1169	0.1887	0.2540
	*Heating oil*		
HAR-RV vs. HAR-RV-US/q = 0.95	0.0027	0.0089	0.0017
HAR-RV vs. HAR-RV-US/q = 0.75	0.0018	0.0006	0.0005
HAR-RV vs. HAR-RV-US/q = 0.5	0.0001	0.0002	0.0003
HAR-RV vs. HAR-RV-US/q = 0.25	0.0002	0.0000	0.0001
HAR-RV vs. HAR-RV-US/q = 0.05	0.0092	0.0036	0.0013
HAR-RV vs. HAR-RV-states/q = 0.95	0.1857	0.2080	0.2754
HAR-RV vs. HAR-RV-states/q = 0.75	0.0499	0.0548	0.1373
HAR-RV vs. HAR-RV-states/q = 0.5	0.0199	0.0544	0.1122
HAR-RV vs. HAR-RV-states/q = 0.25	0.0749	0.0969	0.1357
HAR-RV vs. HAR-RV-states/q = 0.05	0.2376	0.2978	0.2995
HAR-RV-US vs. HAR-RV-states/q = 0.95	0.1835	0.2009	0.2742
HAR-RV-US vs. HAR-RV-states/q = 0.75	0.0481	0.0543	0.1369
HAR-RV-US vs. HAR-RV-states/q = 0.5	0.0198	0.0542	0.1119
HAR-RV-US vs. HAR-RV-states/q = 0.25	0.0747	0.0969	0.1356
HAR-RV-US vs. HAR-RV-states/q = 0.05	0.2306	0.2953	0.2986
	*Natural gas*		
HAR-RV vs. HAR-RV-US/q = 0.95	0.0010	0.0015	0.0010
HAR-RV vs. HAR-RV-US/q = 0.75	0.0069	0.0109	0.0068
HAR-RV vs. HAR-RV-US/q = 0.5	0.0056	0.0080	0.0165
HAR-RV vs. HAR-RV-US/q = 0.25	0.0076	0.0111	0.0312
HAR-RV vs. HAR-RV-US/q = 0.05	0.0003	0.0301	0.0679
HAR-RV vs. HAR-RV-states/q = 0.95	0.2017	0.1191	0.2681
HAR-RV vs. HAR-RV-states/q = 0.75	0.0502	0.0715	0.1153
HAR-RV vs. HAR-RV-states/q = 0.5	0.0138	0.0329	0.0904
HAR-RV vs. HAR-RV-states/q = 0.25	0.0454	0.0959	0.1571
HAR-RV vs. HAR-RV-states/q = 0.05	0.1419	0.2592	0.2853
HAR-RV-US vs. HAR-RV-states/q = 0.95	0.2009	0.1178	0.2674
HAR-RV-US vs. HAR-RV-states/q = 0.75	0.0436	0.0612	0.1093
HAR-RV-US vs. HAR-RV-states/q = 0.5	0.0082	0.0251	0.0751
HAR-RV-US vs. HAR-RV-states/q = 0.25	0.0381	0.0857	0.1300
HAR-RV-US vs. HAR-RV-states/q = 0.05	0.1416	0.2362	0.2333

The relative-performance statistic, *RP*, statistic is computed as $RP = 1 - \sum _{t=1}^T \rho _\alpha \left( e_{t, R} \right) / \sum _{t=1}^T \rho _\alpha \left( e_{t, B} \right)$, where $e_t$ denotes the model prediction errors. The benchmark (B) model is the first model given in the first column of the table, and the rival (R) model is the second model given in that column The HAR-RV-states model includes the state-level components in the vector of potential predictors. The benchmark model is estimated by the quantile-regression technique, while the HAR-RV-states model is estimated by the quantile Lasso technique. The intercept and the classic HAR-RV terms are not penalized. The penalty parameter is determined by tenfold cross-validation. A positive *RP* statistic shows that the rival model outperforms the benchmark model. The parameter *h* denotes the forecast horizon. The parameter *q* denotes the quantile being analyzed. The dependent variable is the realized volatility series as provided by Risk Lab (see Footnote 4 for details)

Finally, Table 7 reports the additional results for data on the realized volatility of returns of crude oil, heating oil, and natural gas prices, whereby, instead of daily data to obtain the weekly values of realized volatility, we rely on underlying intraday data for the estimations, because intraday data contains rich information that can lead to more accurate estimates of volatility (McAleer and Medeiros 2008). The daily realized volatility data are derived from Risk Lab.^18^ For our empirical research, we sum-up over a week the daily realized volatility estimates based on 5-min subsampled returns of the NYMEX light crude oil, NYMEX heating oil No. 2, and NYMEX natural gas futures, with the sample period covering the fourth week of December 2000 to the fourth week of December 2021. It is reassuring to observe that our main results also apply not only to the realized volatility of crude oil derived using an alternative approach but also to heating oil and natural gas. In other words, the intuitive explanation of the results provided above based on weekly RV computed from daily data is robust to the use of an alternative data frequency to derive metrics of volatility for oil and the general energy market, which also includes heating oil and natural gas.

## Concluding remarks

We have shown for the U.S. that state-level economic activity as measured has quantile-dependent predictive value for the subsequent realized volatility of oil price returns. While predictability is weak and hardly existent at a weekly horizon, evidence of predictability strengthens at biweekly and monthly horizons. Using the popular HAR-RV model as the starting point of our empirical analysis, we recovered robust evidence that predictability is particularly strong at the upper (95%) and lower (5%) quantiles of the conditional distribution of realized volatility. Given that the U.S. is a major player in the international oil market, and given that the results of much significant earlier empirical research clearly demonstrate that movements in the price of oil predict subsequent macroeconomic fluctuations at business cycle frequencies (Salisu et al. 2021), we believe that the results documented in this research are of paramount importance for policymakers. In addition to the policy implications of our findings, the role of state-level economic conditions in predicting the volatility of oil price returns also assists in the portfolio allocation decisions of oil traders. Finally, we consider our observations to be important from the perspective of academics studying the determinants of fluctuations in oil prices. Our results clearly demonstrate that state-level economic activity, in addition to that associated with the U.S. economy considered as a single entity, should be added to the list of potentially influential determinants of the volatility of oil price returns in future research.

Recent studies by Bouri et al. (2021) and Gupta and Pierdzioch (2021b) highlight the role of global and climate risks of the overall U.S. in predicting oil price return volatility. Given the results documented by these researchers, as part of future research, it would be interesting to compare the relative importance of state-level climate risks with that of the aggregate U.S. in predicting the variability of movements of the price of crude oil, natural gas, and heating oil, because climate risks have been shown to drive state-level economic conditions in the U.S. (Sheng et al. 2022, forthcoming).

## Data Availability

The datasets used and/or analysed during the current study are available from the corresponding author on reasonable request

## Appendix

See Tables 8 and 9.

**Table 8:** Dataset for state-level economic conditions index

Data category	Variables
Mobility	Cellphone mobility index
	Retail gasoline price
	Vehicle miles traveled
Labour market	Initial unemployment insurance claims
	Continued unemployement insurance claims
	Total nonfarm employment
	Unemployment rate
	Average hours worked in manufacturing
Real activity	Coal production
	Oil rig counts
	Oil production
	Electricity consumption
	Real exports of goods
	Real GDP
Expectations	Business applications
	New housing permits
	Consumer sentiment index
	Manufacturing sentiment index
Financials	Manicipal bonds: yield to maturity
	Municipal bonds: performance
	Real trade-weighted value of the dollar
Households	Credit and debit card spending
	Real wage and salary income
	Real home price index

The reader is referred to the working paper version of Baumeister et al. (2022), available at: http://www.nber.org/papers/w29003, for further details on frequency, geographic coverage, starting date, data transformation, data source, and seasonal adjustment

**Table 9:** Dataset for U.S. economic conditions index

Data category	Variables
Mobility	Cellphone mobility index
	Retail gasoline price
	Vehicle miles traveled
Labour market	Initial unemployment insurance claims
	Continued unemployement insurance claims
	Total nonfarm employment
	Unemployment rate
	Average hours worked in manufacturing
Real activity	Coal production
	Oil rig counts
	Oil production
	Electricity consumption
	Real exports of goods
	Industrial production
	Real GDP
Expectations	Business applications
	New housing permits
	University of Michigan: Consumer sentiment
	Business Tendency Survey for Manufacturing
Financials	10-year Treasury yield
	Corporate bond spread: BAA-AAA
	Real trade-weighted value of the dollar
Households	Credit and debit card spending
	Real wage and salary income
	Real home price index

The reader is referred to the working paper version of Baumeister et al. (2022), available at: http://www.nber.org/papers/w29003, for further details on frequency, starting date, data transformation, data source, and seasonal adjustment

## Abbreviations

ECI: Economic conditions index
EIA: U.S. Energy Information Administration
GARCH: Generalized autoregressive conditional heteroskedasticity
GECON Index: Global Economic Conditions Index
GFC: Global financial crisis
GDP: Real gross domestic product
HAR-RV model: Heterogeneous autoregressive realized volatility model
Lasso estimator: Least absolute shrinkage and selection estimator
MIDAS: Mixed data sampling
RP statistic: Relative performance statistic
RV: Realized volatility
VAR: Vector autoregressive
WTI: West Texas Intermediate

## Footnotes

1 Accurate predictions of the volatility of oil-price returns certainly also can play an important role for managerial decisions on real investment projects. Because a multi-criteria approach is likely to be needed for evaluation of whether such projects are beneficial, our analysis could be combined with, for example, a fuzzy multidimensional decision-making approach (e.g., Kou et al. 2021) to improve decision making. Furthermore, predictions of the volatility of oil-price returns may also help to contribute to asses, for example, crash risk in stock markets (e.g., Wen et al. 2019), given that it is well-known that developments in oil and stock markets are linked.

2 See, for example, Bonaccolto et al. (2018), Asai et al. (2019, 2020), Bonato et al. (2020), Bouri et al. (2020), Demirer et al. (2020, 2021), Gkillas et al. (2020a), Gupta and Pierdzioch (2021a, 2022), Luo et al. (2022), Salisu et al. (forthcoming), and the references cited within these papers.

3 Baumeister et al. (2020) find that the GECON index can be used to accurately forecast oil price returns based on vector autoregressive (VAR) models traditionally used in the modeling of oil price and/or returns movements. Lv and Wu (2022) confirm this finding in a predictive-regression set-up with controls that relate to stock returns forecasting, in light of the close linkage between oil and stock markets.

4 The GECON index comprises real economic activity, commodity (excluding precious metals and energy) prices, financial indicators, transportation, uncertainty, expectations, weather, and energy market-related indicators.

5 The other predictors that Guo et al. (2022), Salisu et al. (2022), and Wang et al. (2022) consider are a real commodity price factor, a global steel-production factor, a real-shipping cost factor, a single-voyage dry-cargo-freight-rates factor, and industrial production of the Organisation for Economic Co-operation and Development (OECD) and six emerging market economies (Brazil, China, India, Indonesia, Russia, and South Africa).

6 The dimensions are the following: Mobility measures, labor market indicators, real economic activity, expectations measures, financial indicators, and household indicators.

7 See the discussions in Chan and Grant (2016) and Lux et al. (2016) for further details.

8 https://www.eia.gov/dnav/pet/hist/RWTCD.htm. The data are accessed from this source on the 23rd of January, 2022.

9 When studying the autocorrelation function plotted in Fig. 1 one should bear in mind that the HAR-RV model aggregates volatilities of different time resolutions into a stylized unified model. In this way, the model captures the long-memory characteristic of the realized volatility of many financial returns series. This aggregation can be interpreted, in economic terms, to reflect the plausible assumption that commodity markets are populated by short-term and long-term traders, with the two groups of market participants responding differently to information flows at different time horizons (see also " Methods" section). The results of the numerical simulations (not reported, but available from the authors upon request) confirmed that the HAR-RV model is consistent with the shape of the autocorrelation function plotted in Fig. 1.

10 The data is publicly available from the Datasets segment of the website of Professor Christiane Baumeister at: https://sites.google.com/site/cjsbaumeister/datasets?authuser=0. The data was accessed from this source on the 23rd of January, 2022.

11 As pointed out in "Data" section, we work with the log of realized volatility; thus, *RV* is to be considered as the log of realized volatility in all equations.

12 It also would be interesting to analyze whether the state-level ECI data can be structured using, e.g., the approach recently developed by Li et al. (2021) and then to analyze whether predictability differs across the members of the clusters computed in this way. We leave a closer analysis of this approach to future research.

13 Two things should be noted. First, we use the relative performance statistic to measure the relative predictive value of the benchmark and the rival model in an in-sample and an out-of-sample context. Second, the HAR-RV model is a nested version of the HAR-RV-US and HAR-RV-states models, but the HAR-RV-US model is not a nested version of the HAR-RV-states model as long as the aggregate economic conditions are not a perfect linear combination of the state-level economic conditions selected by the quantile Lasso estimator.

14 The results summarized in Table 1 are unchanged qualitatively when we study an adjusted relative performance statistic that accounts for the fact that the HAR-RV-states model features a larger number of estimated parameters than the other two models (unless the Lasso estimator sets the coefficients of all state-level economic conditions to zero). We compute the adjusted relative-performance statistic as $RP = 1 - \left[ \sum _{t=1}^T \rho _\alpha \left( e_{t, R} \right) / \sum _{t=1}^T \rho _\alpha \left( e_{t, B} \right) \right] \left[ ( T - P_B ) / ( T - P_R ) \right]$, where $P_B$ ($P_R$) denotes the number of parameters of the benchmark and rival model (that is, the number of non-zero coefficients of the HAR-RV-states model under the quantile Lasso estimator). The term $( T - P_B ) / ( T - P_R )$ reduces relative performance when the rival model features more coefficients than the benchmark model. Results for the adjusted relative performance statistic are not reported for the sake of brevity, but available from the authors upon request.

15 When we study realized volatility at the end of the forecast horizon rather than average realized volatility over the forecast horizon, we again observe that the HAR-RV-US model does not add much value relative to the HAR-RV model. The HAR-RV-states model, while its relative performance statistics as excepted tend to be smaller than the statistics reported Table 1, continues to have a discernible impact on forecasting performance relative to the HAR-RV and HAR-RV-US model at the biweekly and monthly forecast horizon. At the monthly horizon, relative performance statistics for the HAR-RV-states model are largest at the median of the conditional distribution of realized volatility. Detailed results are available from the authors upon request.

16 The data can be downloaded from: https://sites.google.com/view/weeklystateindexes/decomposition?authuser=0, and basically captures the historical decomposition of the economic conditions indexes of each state. The data was accessed from this source on the 23rd of January, 2022.

17 The data is downloadable from: https://sites.google.com/view/weeklystateindexes/economic-weakness-index?authuser=0. The data was accessed from this source on the 23rd of January, 2022.

18 Risk Lab is maintained by Professor Dacheng Xiu at Booth School of Business, University of Chicago. The data is downloadable from the following internet page: https://dachxiu.chicagobooth.edu/#risklab. Note that the data was accessed from this source on the 23rd of January, 2022. As described in detail on this internet page, estimates of realized volatility are based on data on trades as collected at the highest frequencies available, where the data are cleared based on the available national best bid and offer. Realized volatility then is computed based on quasi-maximum likelihood estimates, building on moving-average models, where non-zero returns of transaction prices are sampled up to their highest frequency available (considering days with at least 12 observations).
